# MIND diet and the risk of dementia: a population-based study

**DOI:** 10.1186/s13195-022-00957-1

**Published:** 2022-01-12

**Authors:** Tosca O. E. de Crom, Sanne S. Mooldijk, M. Kamran Ikram, M. Arfan Ikram, Trudy Voortman

**Affiliations:** 1 Department of Epidemiology, Erasmus MC, University Medical Center, PO Box 2040, 3000 CA Rotterdam, The Netherlands; 2 Department of Neurology, Erasmus MC, University Medical Center, Rotterdam, The Netherlands; 3 Division of Human Nutrition and Health, Wageningen University & Research, Wageningen, The Netherlands

**Keywords:** MIND diet, Dietary pattern, Dementia, Population-based, Epidemiology

## Abstract

**Background:**

Adherence to the Mediterranean-DASH Intervention for Neurodegenerative Delay (MIND) diet has been linked to a decreased risk of dementia, but reverse causality and residual confounding by lifestyle may partly account for this link. We aimed to address these issues by studying the associations over cumulative time periods, which may provide insight into possible reverse causality, and by using both historical and more contemporary dietary data as this could give insight into confounding since historical data may be less affected by lifestyle factors.

**Methods:**

In the population-based Rotterdam Study, dietary intake was assessed using validated food frequency questionnaires in 5375 participants between 1989 and 1993 (baseline I) and in a largely non-overlapping sample in 2861 participants between 2009 and 2013 (baseline II). We calculated the MIND diet score and studied its association with the risk of all-cause dementia, using Cox models. Incident all-cause dementia was recorded until 2018.

**Results:**

During a mean follow-up of 15.6 years from baseline I, 1188 participants developed dementia. A higher MIND diet score at baseline I was associated with a lower risk of dementia over the first 7 years of follow-up (hazard ratio (HR) [95% confidence interval (CI)] per standard deviation (SD) increase, 0.85 [0.74, 0.98]), but associations disappeared over longer follow-up intervals. The mean follow-up from baseline II was 5.9 years during which 248 participants developed dementia. A higher MIND diet score at baseline II was associated with a lower risk of dementia over every follow-up interval, but associations slightly attenuated over time (HR [95% CI] for 7 years follow-up per SD increase, 0.76 [0.66, 0.87]). The MIND diet score at baseline II was more strongly associated with the risk of dementia than the MIND diet score at baseline I.

**Conclusion:**

Better adherence to the MIND diet is associated with a decreased risk of dementia within the first years of follow-up, but this may in part be explained by reverse causality and residual confounding by lifestyle. Further research is needed to unravel to which extent the MIND diet may affect the risk of dementia.

**Supplementary Information:**

The online version contains supplementary material available at 10.1186/s13195-022-00957-1.

## Introduction

Diet has gained increasing interest as a target for developing preventive opportunities against dementia, as it impacts several mechanisms underlying dementia, including oxidative stress, inflammation, and vascular abnormalities. Accordingly, various studies have linked adherence to healthy dietary patterns to a slower rate of cognitive decline [1] and a decreased risk of dementia [2]. Although such healthy dietary patterns may be sub-optimal for brain health. Therefore, the Mediterranean-Dietary Approaches to Stop Hypertension (DASH) Intervention for Neurodegenerative Delay (MIND) diet has been developed [3], to uniquely emphasize foods linked to brain health, such as green leafy vegetables [4, 5] and berries [6]. Adherence to the MIND diet has indeed been linked to better cognitive performance [7, 8], less cognitive decline [3, 9–11], and a lower dementia risk [12, 13]. Nevertheless, in observational studies linking dietary patterns to dementia, two methodological issues remain challenging to address.

First, the average follow-up time in previous studies was 4.5 to 6.6 years, which corresponds largely to the prodromal stage of dementia [14]. During this phase of incremental cognitive impairment, dietary habits may deteriorate [15, 16], for instance, due to depressive symptoms [17] and olfactory impairment [18]. This may introduce reverse causality in short-term associations between dietary patterns and incident dementia. Studies with long follow-ups allowing for studying association over cumulative time periods could provide insights into possible reverse causality.

Second, studying dietary patterns in observational studies is notoriously difficult since it invariably suffers from confounding by lifestyle. Although previous studies controlled for lifestyle factors in their analyses, residual confounding may persist [19, 20]. In this regard, it is important to realize that healthy diet and lifestyle awareness increased steeply over the past few decades and most likely thereby also the relation between diet quality and other lifestyle factors [21]. Meaning that those who care most about their health adhere to both a healthy diet and lifestyle, while those who care less about their health adhere to a less healthy diet and lifestyle. Using both historical and more contemporary data on dietary patterns in the same study population and comparing their association with the risk of dementia may give insight in confounding since historical data may be less affected by lifestyle factors than more contemporary data.

The Rotterdam Study is a prospective population-based study, with dietary intake assessed in 1989–1993 and again in 2009–2013 in a largely non-overlapping sample. We determined the association between adherence to the MIND diet and the risk of dementia across these two settings two decades apart, over cumulative follow-up intervals. Moreover, to unravel whether the MIND diet is indeed more specific for brain health than other healthy diets, we also studied the association between adherence to two other healthy diets and the risk of dementia.

## Method

### Study population

This study was embedded within the first two sub-cohorts of the Rotterdam Study (RS), a prospective population-based cohort among inhabitants from the suburb Ommoord in Rotterdam, the Netherlands. Details regarding the study methodology have been published previously [22]. Briefly, the first sub-cohort (RS-I) was established in 1989 and consisted of 7983 participants aged 55 years and older. In 1999, the study was expanded with the second sub-cohort (RS-II) consisting of 3011 participants who had turned 55 years of age or moved into the study area. Extensive follow-up examination rounds take place every 3–5 years through home interviews and various physical and laboratory checks at the research center.

For the current study, we consider two different baselines. The periods considered as baselines were dependent on the examination rounds in which dietary intake was assessed: between 1989 and 1993 in the first cohort (RS-I-1), which forms baseline I in the current study, and between 2009 and 2012 in the first and second cohort (RS-I-5 and RS-II-3), which forms baseline II in the current study.

Of the 7983 participants included in the study at baseline I, 5435 participants had dietary data available. We excluded 3 participants who had unreliable dietary data (i.e., an estimated energy intake of < 500 or > 5000 kcal/day), 22 participants who had dementia at the time of dietary assessment, and 35 participants who did not the sign informed consent to link the study database to their medical records. This leaves a total of 5375 participants eligible for follow-up from baseline I. Of the 4040 participants who participated at baseline II, 2998 participants had dietary data available. We excluded 110 participants who had unreliable dietary data, 23 participants who had dementia at the time of dietary assessment, 1 participant who had insufficient cognitive screening to assess dementia, and 3 participants who did not sign informed consent to link the study database to their medical records. This leaves a total of 2861 participants eligible for follow-up from baseline II. A schematic overview of the study population is provided in Additional file 1.

### Dietary assessment

At baseline I, participants completed a 170-item food frequency questionnaire (FFQ). They first completed a checklist on which food items they consumed at least twice a month in the preceding year, after which information on frequencies and portion sizes was obtained in an interview by a trained dietician. At baseline II, dietary intake was assessed with a self-administered 389-item FFQ including questions on frequency and portion sizes of food item consumption in the last month. Both FFQs have been validated against other dietary assessment methods which showed that based on these FFQs, participants can be adequately ranked according to their food and nutrient intake [23–25]. From the FFQ data, we derived adherence scores for the MIND diet, Dutch dietary guidelines, and Mediterranean diet, as outlined below.

### MIND diet

The MIND diet as described by Morris et al. [3] contains recommendations regarding 15 food components, including 10 food components considered to be healthy for the brain (i.e., green leafy vegetables, other vegetables, nuts, berries, beans, whole grains, fish, poultry, olive oil, and wine) and five unhealthy food components (i.e., red meat, butter and stick margarine, cheese, fast fried food, and pastries and sweets). An overview of food items on the different FFQs that summarizes these food components can be found in Additional file 1. If participants used olive oil as the primary cooking fat (> 50%), a 1 was assigned and a 0 otherwise. For each other food component, a 0 was assigned if participants did not adhere to the recommendations, a 0.5 for moderate adherence, and a 1 for good adherence. Scores assigned to each food component were summed, obtaining a total score ranging from 0 to 15.

### Dutch dietary guidelines

We used a previously defined score to assess adherence to Dutch dietary guidelines [26]. Briefly, participants received a score of 1 (adherence) or 0 (no adherence) for recommendations of 14 food components (i.e., vegetables, nuts, fruits, legumes, whole grains, whole grains of total grains, fish, dairy products, tea, coffee, unsaturated fats and oils of total fats, red and processed meat, sugar-containing beverages, alcoholic beverages, and salt). The sum score ranged from 0 to 14.

### Mediterranean diet

The Mediterranean diet is described by Panagiotakos et al. [27] containing recommendations regarding 11 food components (i.e., vegetables, fruits, legumes, whole grains, fish, full-fat dairy products, potatoes, olive oil, poultry, meat, and alcoholic beverages). Adherence was determined by assigning a score ranging from 0 to 5 to each food component with higher scores reflecting better adherence. The final sum score ranged from 0 to 55.

### Dementia

Participants were screened for dementia at baseline and every 3–5 years during follow-up examinations using the Mini-Mental State Examination (MMSE) and the Geriatric Mental Schedule (GMS) organic level. Those with an MMSE score of < 26 or a GMS organic level score of > 0 were further examined using the Cambridge Examination for Mental Disorders in the Elderly diagnostic interview. Additionally, participants were continuously under surveillance for dementia through the electronic linkage between the study database and medical records from general practitioners and the Regional Institute of Outpatients Mental health Care. The final diagnosis of dementia and its most common subtypes was made by a consensus panel led by a neurologist based on the standard criteria for all-cause dementia (DSM-III-R) and for sub-diagnosis of Alzheimer’s disease (NINCDS-ADRDA). Follow-up for dementia was completed until January 1, 2018.

### Covariates

Data on relevant covariates were obtained at both baselines I and II. Trained interviewers obtained information regarding education attainment (primary, lower, intermediate, higher), smoking status (never, former, current), and use of medication. Height and weight were measured, and body mass index (BMI) (kg/m^2^) was calculated. Physical activity was measured using a validated adapted version of the Zutphen Physical Activity Questionnaire at baseline I and the LASA Physical Activity Questionnaire at baseline II. Physical activity was expressed in metabolic equivalent of task (MET)—hours per week. Daily energy intake in kilocalories was calculated from the FFQ data using the Dutch Food Composition Tables (NEVO). Diabetes mellitus was defined as having a fasting serum glucose of ≥ 7.0 mmol/L, a random serum glucose level of ≥ 11.1 mmol/L, or the use of blood glucose-lowering medication. Hypercholesterolemia was defined as a serum total cholesterol concentration ≥ 6.2 mmol/L or the use of lipid-lowering medication. Systolic and diastolic blood pressure was measured twice on the right arm with the participant in a sitting position using a random zero sphygmomanometer of which the mean was used for analyses. Hypertension was defined as a systolic blood pressure of ≥ 140 mmHg, a diastolic blood pressure of ≥ 90 mmHg, or the use of blood pressure-lowering medication. Depressive symptoms were considered as a score of ≥ 16 on the validated Center for Epidemiology Depression Scale. History of stroke was obtained from interviews and verified through medical records. *APOE* genotype was obtained using polymerase chain reaction of coded DNA samples for RS-I and with bi-allelic TaqMan assay for RS-II.

### Statistical analysis

Cox proportional hazard models were used to determine the association between the different diet scores per standard deviation (SD) increase and incidence all-cause dementia. Analyses were conducted from baselines I and II separately. Participants were censored when they were diagnosed with dementia, died, were lost to follow-up, or at the end of the follow-up (January 1, 2018), whichever came first. To test for potential non-linear relationships, we added natural cubic splines with three knots to the diet scores in het model and tested whether this significantly improved the fit of the model using likelihood ratio tests. To determine how associations changed over time, we performed analyses in cumulative follow-up intervals from the different baselines (i.e., performing analyses from the different baselines to 5 years, baselines to 7 years) [28]. We constructed a basic model adjusted for sex, age, age^2, and educational attainment (model 1). Subsequently, we further adjusted for smoking status, physical activity, and energy intake (model 2). To minimize the risk of residual confounding, we considered an additional model in which we further adjusted for covariates that may act as confounders and/or mediators, which include BMI, diabetes, hypercholesterolemia, and hypertension (model 3). Missing data on covariates (29% for physical activity and < 5% for all other covariates) were imputed using five-fold multiple imputation. The distribution of the covariates in the imputed dataset was comparable to the original dataset (data not shown). Analyses were performed on each imputed dataset, and the results were presented as pooled hazard ratios (HRs) with 95% confidence intervals (95% CIs). All analyses are repeated considering Alzheimer’s dementia as the outcome variable. Possible effect modification by sex, educational attainment, smoking status, and *APOE* ε4 genotype (carrier vs. non-carrier) was investigated by including multiplicative interaction terms to the MIND diet score in model 2, and if the interaction term was statistically significant (*p* < 0.05), we performed stratified analyses.

To examine if single food components of the MIND diet drove the observed associations, we repeated the analyses with versions of the MIND diet score for which each individual food component was one at a time excluded from the total score and included as a covariate in the het model. Furthermore, as the MIND diet covers five unhealthy food components which are not covered in the Dutch dietary guidelines and Mediterranean diet, we excluded all unhealthy food components altogether from the total score and included these five components as a covariate in the model to determine whether these unhealthy food components together drove the association.

To ensure the robustness of our findings, we conducted several sensitivity analyses. First, we repeated the analyses for participants above and below the age of 75 years separately. Second, as cognitive impairment might have influenced the reliability of dietary recall, we repeated the analyses after excluding participants with an MMSE score of < 26 at the time of dietary assessment. Third, participants with a history of stroke at dietary assessment were excluded and censored at the date of incidence of stroke. Fourth, having depressive symptoms is an important confounder in the association between dietary intake and dementia risk, but we had no data on depressive symptoms for 58.2% of the participants. We therefore repeated the analyses after excluding all participants with depressive symptoms or missing data on depressive symptoms. Finally, we repeated the analyses after excluding the first 5 years of follow-up, to assess potential reverse causality.

All statistical analyses were conducted using the R Statistical Software version 4.0.3.

## Results

Characteristics of the study population at baselines I and II are presented in Table 1. In Additional file 1, characteristics of the study population are also presented stratified by tertiles of the MIND diet score. Participants at baseline II were on average older, higher educated, less often current smokers, and less physically active than participants at baseline I. Additional file 1 provides the baseline characteristics stratified by age above and below 75 years and shows that differences in the physical activity levels between the baselines are mainly attributable to age.

**Table 1:** Characteristics of the study population at baselines I and II

	Baseline I (between 1989 and 1993) (***N*** = 5375)	Baseline II (between 2009 and 2012) (***N*** = 2861)
**Sex** (women)	3169 (59.0)	1643 (57.4)
**Age** (years)	67.7 ± 7.8	75.3 ± 5.9
**Education attainment**		
Primary	1102 (20.6)	192 (6.8)
Lower	2281 (42.6)	1215 (43.3)
Intermediate	1504 (28.1)	930 (33.1)
Higher	463 (8.7)	469 (16.7)
**Smoking status**		
Never	1800 (33.7)	914 (32.0)
Former	2296 (43.0)	1667 (58.4)
Current	1247 (23.3)	274 (9.6)
**Body mass index** (kg/m^2^)	26.3 ± 3.6	27.4 ± 4.2
**Physical activity** (MET h/week)	83.4 ± 43.9	50.0 ± 45.4
**Daily energy intake** (kcal)	1974 ± 499	1994 ± 658
**Diabetes** (yes)	357 (6.9)	418 (15.2)
**Hypercholesterolemia** (yes)	3602 (67.1)	1549 (56.1)
**Hypertension** (yes)	3202 (59.8)	2461 (87.0)
**Depressive symptoms** (yes)	314 (18.7)	713 (15.7)
**History of stroke** (yes)	98 (1.8)	145 (5.1)
***APOE*** **ɛ4 carrier** (yes)	1416 (27.5)	703 (26.1)
**Diet scores, mean ± standard deviation (range)**		
MIND diet	5.9 ± 1.3 (2.0–11.5)	7.4 ± 1.6 (1.5–13.5)
Dutch dietary guidelines	6.8 ± 1.8 (1–13)	6.8 ± 1.8 (1–13)
Mediterranean diet	36.7 ± 3.3 (20–47)	37.1 ± 4.1 (19–51)

Data are shown for non-imputed data and are presented as mean ± standard deviation for continuous variables and number (percentages) for categorical variables unless stated otherwise

*Abbreviations*: *APOE* Apolipoprotein E, *MET* Metabolic equivalent of task, *MIND* Mediterranean-Dietary Approaches to Stop Hypertension Intervention for Neurodegenerative Delay; *N*, total number of participants

Participants’ mean (SD) MIND diet score was 5.9 (1.3) at baseline I and 7.4 (1.6) at baseline II on a theoretical range from 0 (no adherence) to 15 (full adherence). Adherence scores of the individual food components are presented in Additional file 1. Participants at baseline I had on average higher adherence scores for berries, beans, and fish and lower adherence scores for fried foods than participants at baseline II. A total of 1244 participants were both included in baselines I and II and therefore had two dietary assessments available. The Pearson correlation coefficient between the first and second MIND diet score was 0.25. Moreover, the MIND diet score was moderately correlated with the Dutch dietary guidelines score (*r* = 0.42 at baseline I and *r* = 0.51 at baseline II) and Mediterranean diet score (*r* = 0.46 at baseline I and *r* = 0.55 at baseline II).

The mean follow-up (range) from baseline I was 15.6 years (0.0–27.7), during which 1188 participants developed all-cause dementia (incidence rate 14 per 1000 person-years). When considering the overall follow-up time, the MIND diet score at baseline I was not associated with the risk of dementia (model 2 adjusted HR [95% CI] per SD increase, 1.00 [0.94, 1.06]), neither were the Dutch dietary guidelines and Mediterranean diet score (Table 2). The mean follow-up (range) from baseline 2 was 5.9 years (0.0–9.1), during which 248 participants developed dementia (incidence rate 15 per 1000 person-years). A higher MIND diet score at baseline II was associated with a lower risk of dementia (model 2 adjusted HR [95% CI] per SD increase, 0.80 [0.70, 0.91]) and so were the Dutch dietary guidelines (model 2 adjusted HR [95% CI] per SD increase, 0.90 [0.79, 1.02]) and Mediterranean diet score (model 2 adjusted HR [95% CI] per SD increase, 0.76 [0.66, 0.86]). Additional adjustment for covariates that may be confounders or mediators in this association (model 3) did not substantially alter the risk estimates. No evidence was found for non-linear associations between the diet scores at either baseline I or II and the risk of dementia (*p* > 0.05).

**Table 2:** Diet scores in association with the risk of all-cause dementia

	***n***/***N***	Hazard ratio (95% confidence interval)		
		Model 1	Model 2	Model 3
**Baseline I (between 1989 and 1993)**	1188/5375			
MIND diet score		0.99 (0.93–1.05)	1.00 (0.94–1.06)	0.99 (0.94–1.05)
Dutch dietary guidelines score		1.01 (0.95–1.07)	1.01 (0.96–1.07)	1.01 (0.96–1.07)
Mediterranean diet score		1.03 (0.97–1.10)	1.04 (0.98–1.10)	1.04 (0.97–1.10)
**Baseline II (between 2009 and 2012)**	248/2861			
MIND diet score		0.79 (0.70–0.90)	0.80 (0.70–0.91)	0.79 (0.70–0.91)
Dutch dietary guidelines score		0.88 (0.78–1.00)	0.90 (0.79–1.02)	0.89 (0.78–1.02)
Mediterranean diet score		0.76 (0.67–0.86)	0.76 (0.66–0.86)	0.75 (0.66–0.86)

Hazard ratios are per standard deviation increase in diet score. Model 1 is adjusted for sex, age, age^2, and educational attainment. Model 2 is further adjusted for smoking status, physical activity, and daily energy intake. Model 3 is further adjusted for body mass index, diabetes, hypercholesterolemia, and hypertension

*Abbreviations*: *MIND* Mediterranean-Dietary Approaches to Stop Hypertension Intervention for Neurodegenerative Delay, *n* number of participants with incident all-cause dementia; *N*, total number of participants

**Fig. 1:**
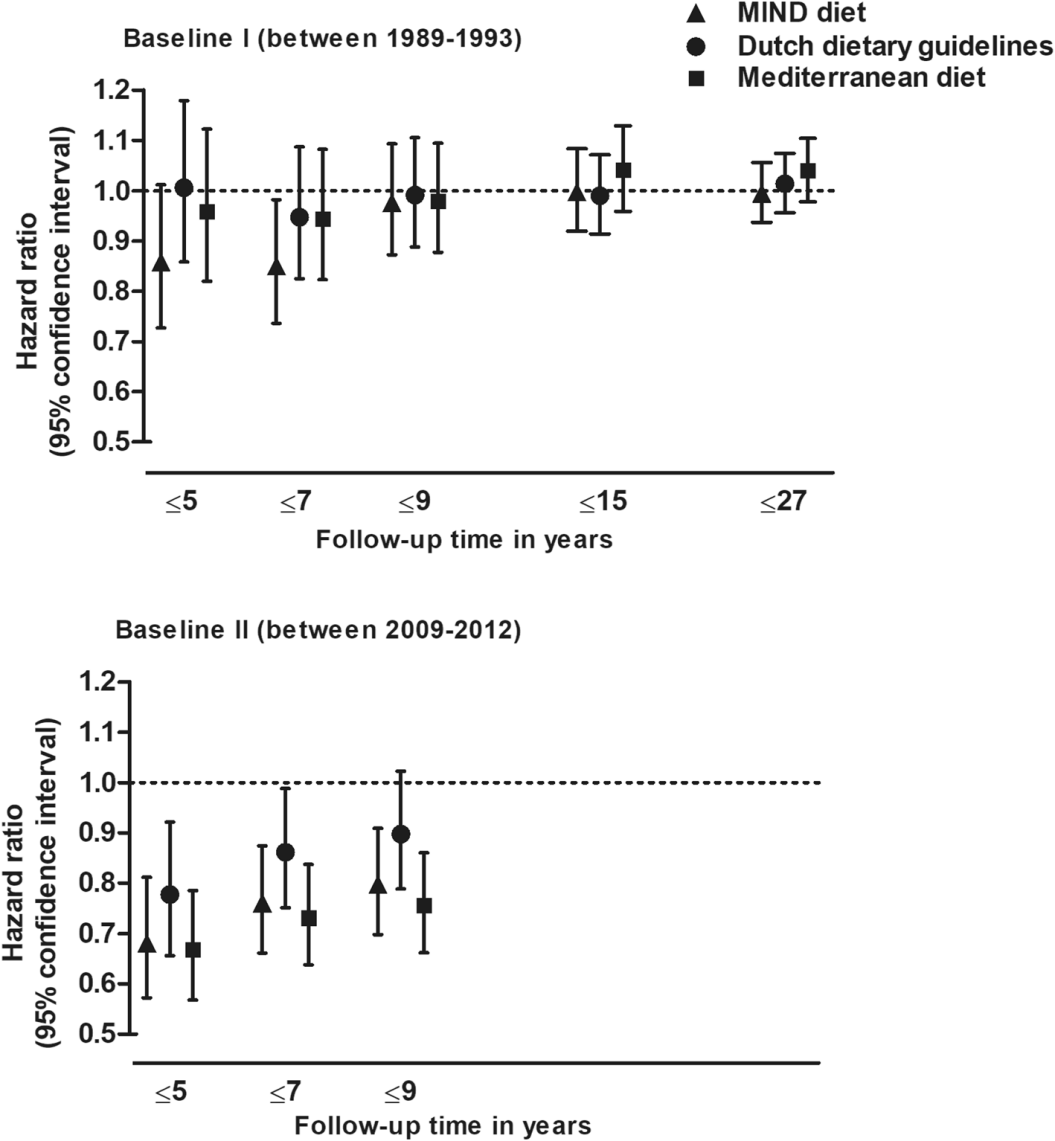
Diet scores in association with the risk of all-cause dementia, per cumulative follow-up interval. Hazard ratios are per standard deviation increase in diet score and adjusted for sex, age, age^2, educational attainment, smoking status, physical activity, and daily energy intake. MIND, Mediterranean-Dietary Approaches to Stop Hypertension Intervention for Neurodegenerative Delay

When analyzing the cumulative follow-up intervals from baseline I, a higher MIND diet score was associated with a lower dementia risk over 5 and 7 years of follow-up, but associations were no longer present over longer follow-up periods (Fig. 1). The Dutch dietary guidelines and Mediterranean diet score at baseline I were not associated with the risk of dementia during any cumulative follow-up interval. From baseline II, a higher MIND diet score was associated with a lower risk of dementia during each cumulative follow-up interval, but risk estimates were strongest within the first 5 years of follow-up and slightly attenuated over longer follow-up periods. Higher Dutch dietary guidelines and Mediterranean diet scores at baseline II were also associated with a lower risk of dementia during every cumulative follow-up interval, and risk estimates were also slightly attenuated over time.

**Fig. 2:**
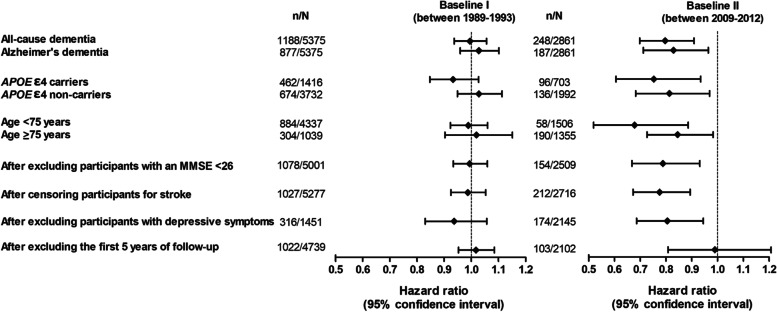
Subgroup and sensitivity analyses for the MIND diet score in association with the risk of all-cause dementia. Hazard ratios are per standard deviation increase in diet score and adjusted for sex, age, age^2, educational attainment, smoking status, physical activity, and daily energy intake. *p* for *APOE* ɛ 4 carrier status interaction 0.108 for baseline I and 0.005 for baseline II. *APOE*, apolipoprotein E; MIND, Mediterranean-Dietary Approaches to Stop Hypertension Intervention for Neurodegenerative Delay; MMSE, Mini-Mental State Examination; *n*, number of participants with incident dementia; *N*, total number of participants

Associations between the MIND diet score and Alzheimer’s dementia were similar as for all-cause dementia (Fig. 2). Effect estimates were somewhat larger in *APOE* ε4 carriers compared to non-carriers (*p* for interaction 0.108 for baseline I and 0.005 for baseline II). From baseline II, the effect estimates were slightly larger in participants aged < 75 years compared to those aged ≥ 75 years, but associations from baseline I were similar between these age groups. No meaningful differences in the risk estimates were observed after excluding participants with an MMSE score of < 26, after excluding participants with a history of stroke and while censoring during follow-up at the data of incidence stroke, and after excluding participants who had depressive symptoms or missing data on depressive symptoms. In line with our findings stratified by follow-up time, associations were no longer present after excluding the first 5 years of follow-up. Moreover, associations were not driven by one individual food component or by the five unhealthy food components altogether (data not shown), and there was no evidence for effect modification by sex, educational attainment, or smoking status (*p* for interaction > 0.05).

## Discussion

In this population-based study, better MIND diet adherence between 1989 and 1993 was associated with a lower risk of dementia over the first 7 years of follow-up, but associations disappeared after longer follow-up periods. Whereas better MIND diet adherence between 2009 and 2021 was associated with a lower risk of dementia over every cumulative follow-up period (maximum of 9 years), but risk estimates were slightly attenuated over time. Besides, MIND diet adherence between 2009 and 2012 was when comparing similar follow-up intervals, more strongly associated with the risk of all-cause dementia than MIND diet adherence between 1989 and 1993. Compared to other healthy diets, associations of the MIND diet were stronger or similar.

Few previous longitudinal studies reported on MIND diet adherence in association with cognitive performance [7, 8], cognitive decline [3, 7–11], or dementia risk [12, 13]. While some studies found an association between better MIND diet adherence and less cognitive decline [3, 9–11], others only observed an association with cognitive performance at baseline [7, 8]. Furthermore, the Memory and Aging Project found that better MIND diet adherence was associated with a lower risk of Alzheimer’s dementia over an average period of 4.5 years (maximum of 9 years) [12]. In the Personality and Total Health Through Life Study, persons who better adhered to the MIND diet were less likely to have all-cause dementia or mild cognitive impairment 12 years after baseline [13]. Our findings of an association within the first years of follow-up build on results from these previous studies, but associations in our study attenuated over time and disappeared after long-term follow-up periods. Studies have shown that dietary habits deteriorate up to 5 years before dementia diagnosis [15, 16] due to prodromal dementia symptoms such as depressive symptoms [17] and olfactory impairment [18]. This implies that when restricting follow-up time to 5 years, incident cases underwent dietary assessment while dietary habits have deteriorated. This could suggest that our findings of attenuating effect estimates may in part be explained by reverse causality.

Observational studies on diet and dementia are prone to residual confounding by lifestyle as individuals with a healthy diet are likely to also lead an overall healthy lifestyle [19, 20], such as meeting recommendations of physical activity and sleep, having lifelong cognitive training and sufficient levels of social contact, and timely visiting general practitioners by adverse health outcomes. Such behavioral factors are challenging to fully control for as data on such confounders is often not collected, impossible to measure, or imprecisely measured (i.e., self-reported or categorized) [19, 20]. As research and communication on the importance of healthy nutrition and lifestyle have strongly increased over the past few decades and thereby healthy diet and lifestyle awareness in the general population [21], we speculate that the relation between adherence to the MIND diet and an overall healthy lifestyle has become stronger over time. Meaning that individuals who care most about their health adhere to a healthy diet and lifestyle, and those who care less about their health adhere to a less healthy diet and lifestyle. Against this background, we used historical and more contemporary dietary data to determine the risk of dementia and found a substantially stronger association when considering MIND diet adherence measured in more contemporary years (1989–1993 vs. 2009–2012). This may suggest that residual confounding by lifestyle explains these discrepancies.

Nevertheless, we do not rule out that observed attenuating risk estimates and discrepancies in the strength of associations across different baselines can be explained by other factors than reverse causality and residual confounding. A potential alternative explanation may be changes in dietary habits independent of the prodromal dementia phase. This could for instance be due to the greater diversity and more affordable prices of both healthy and unhealthy products and less seasonal dependence [29]. Indeed, the correlation of the MIND diet score after 20 years of follow-up was relatively low (*r* = 0.25). Besides, participants had between 1989 and 1993 an average MIND diet score of 5.9, which is relatively low compared to the average score of 7.4 that participants had between 2009 and 2012. This difference is mainly attributable to higher consumption of berries, beans, fish, and fried food between 2009 and 2012. The average MIND diet score between 1989 and 1993 was also relatively low compared to the average scores from previous studies that ranged from 6.3 to 9.4, although most previous studies did not report on average individual food components scores, limiting direct comparisons with our study. Another alternative explanation for discrepancies in the strength of associations across baselines can be differences in population characteristics. Participants between 2009 and 2012 were generally older, higher educated, less often smokers, and less physically active than participants between 1989 and 1993, although differences in physical activity levels are mainly attributable to the difference in age and we did not observe major differences in effect estimates after repeating the analyses for participants above and below the age of 75 years. Also, we did not find evidence for effect modification by educational attainment or smoking status. We therefore assume that observed discrepancies across the baselines cannot be explained by the differences in these lifestyle factors.

In line with other studies [3, 10, 12, 13], we found that associations for the MIND diet were stronger or similar compared to associations for other healthy diets, which support an effect of the MIND diet on the risk of dementia. The MIND diet emphasizes several food components, among which uniquely green leafy vegetables and berries, that could protect against dementia through their anti-inflammatory and ant-oxidative capacity [4–6]. Moreover, the MIND diet may protect against dementia through its favorable effects on cardiovascular risk factors such as obesity, insulin resistance, hypercholesterolemia, and hypertension [30, 31]. To further elucidate to what extent the MIND diet is accountable for the risk of dementia, we encourage future studies to compare long-term trajectories of adherence to the MIND diet before dementia diagnoses to trajectories of healthy controls, to link long-term MIND diet adherence to pre-clinical markers of dementia, and to conduct intervention studies.

### Limitations

Strengths of our study include its prospective population-based design with dietary intake measurements between 1989 and 1993 as well as between 2009 and 2013, and the long follow-up for incidence of dementia. This unique combination of data allowed us to link MIND diet adherence at both periods to the risk of dementia over cumulative follow-up periods. We could thereby create more insight into whether reverse causality and residual confounding by lifestyle modify the association. However, there are some limitations that should be taken into account when interpreting our results. First, different FFQs that varied in the number of items were used to determine dietary intake at baselines I and II, which complicates the direct comparison, although the main difference between the FFQs was the level of detail on the food items, rather than the food items themselves. Only the items that summarize the components “fried foods” and “pastries and sweets” varied substantially between baselines I and II, but we found no major differences in effect estimates after repeating the analyses with versions of the MIND diet score from which these food groups were excluded. Also, validation studies have shown that both FFQs can be used to rank participants adequately according to their dietary intake [23–25]. We therefore believe that comparisons of associations between the baselines are reliable. Second, strawberries were the only berries specified under fruit intake in the FFQs, while other berries such as blueberries, blackberries, and raspberries are also included in the MIND diet. Last, dietary habits were self-reported based on FFQs, while participants with cognitive impairment as a result of prodromal dementia may have not been able to recall their dietary habits accurately. Yet, we did not observe meaningful differences in risk estimates after excluding participants with an MMSE score of < 26.

## Conclusion

Better adherence to the MIND diet is associated with a decreased risk of dementia within the first years of follow-up, but this may in part be explained by reverse causality and residual confounding by lifestyle. Further research is needed to unravel to which extent the MIND diet may affect the risk of dementia by for instance focusing on MIND diet adherence trajectories before dementia diagnosis, by studying MIND diet adherence in association with pre-clinical markers of dementia, and by conducting intervention studies.

## Supplementary Information

**Additional file 1: Figure S1.** Schematic overview of eligible Rotterdam Study (RS) participants. **Table S1.** Food items of the food frequency questionnaires (FFQs) that summarize the individual food components emphasized by the MIND diet. **Table S2.** Characteristics of the study population at baseline I and II stratified for MIND diet tertiles. Note: Data are shown for non-imputed data and are presented as mean ± standard deviation for continuous variables and number (percentages) for categorical variables. Abbreviations: *APOE*, apolipoprotein ɛ; MET, Metabolic Equivalent of Task; MIND, Mediterranean- Dietary Approaches to Stop Hypertension Intervention for Neurodegenerative Delay; N, total number of participants. **Table S3.** Characteristics of the study population at baseline I and II stratified for age above and below 75 years. Note: Data are shown for non-imputed data and are presented as mean ± standard deviation for continuous variables and number (percentages) for categorical variables. Abbreviations: *APOE*, apolipoprotein ɛ; MET, Metabolic Equivalent of Task; MIND, Mediterranean- Dietary Approaches to Stop Hypertension Intervention for Neurodegenerative Delay; N, total number of participants. **Table S4.** Adherence scores of the individual food components. Note: If participants used olive oil as primary cooking fat (>50%) a 1 was assigned (good adherence) and a 0 otherwise (no adherence). For each other food component, a 0 was assigned if participants did not adhere to the recommendations, a 0.5 for moderate adherence, and a 1 for good adherence.

## Data Availability

Because of data protection standards of the informed consent procedure of the Rotterdam Study, data cannot be made freely available in publicly available repositories.

## Abbreviations

*APOE*: Apolipoprotein E
BMI: Body mass index
DASH: Dietary Approaches to Stop Hypertension
FFQ: Food frequency questionnaire
GMS: Geriatric Mental Schedule
MET: Metabolic equivalent of task
MMSE: Mini-Mental State Examination
MIND: Mediterranean-Dietary Approaches to Stop Hypertension (DASH) Intervention for Neurodegenerative Delay
RS: Rotterdam Study
SD: Standard deviation
