# Supplementary material for: MIND diet and the risk of dementia: a population-based study
Source: Alzheimers Res Ther. 2022 Jan 12;14:8. doi: 10.1186/s13195-022-00957-1 (PMC8756695; doi:10.1186/s13195-022-00957-1)
Supplement: Supplementary file 1 — Additional file 1: Figure S1. Schematic overview of eligible Rotterdam Study (RS) participants. Table S1. Food items of the food frequency questionnaires (FFQs) that summarize the individual food components emphasized by the MIND diet. Table S2. Characteristics of the study population at baseline I and II stratified for MIND diet tertiles. Note: Data are shown for non-imputed data and are presented as mean ± standard deviation for continuous variables and number (percentages) for categorical variables. Abbreviations: APOE, apolipoprotein ɛ; MET, Metabolic Equivalent of Task; MIND, Mediterranean- Dietary Approaches to Stop Hypertension Intervention for Neurodegenerative Delay; N, total number of participants. Table S3. Characteristics of the study population at baseline I and II stratified for age above and below 75 years. Note: Data are shown for non-imputed data and are presented as mean ± standard deviation for continuous variables and number (percentages) for categorical variables. Abbreviations: APOE, apolipoprotein ɛ; MET, Metabolic Equivalent of Task; MIND, Mediterranean- Dietary Approaches to Stop Hypertension Intervention for Neurodegenerative Delay; N, total number of participants. Table S4. Adherence scores of the individual food components. Note: If participants used olive oil as primary cooking fat (>50%) a 1 was assigned (good adherence) and a 0 otherwise (no adherence). For each other food component, a 0 was assigned if participants did not adhere to the recommendations, a 0.5 for moderate adherence, and a 1 for good adherence. [file 13195_2022_957_MOESM1_ESM.docx]

Additional file

Additional file 1: Figure S1. Schematic overview of eligible Rotterdam Study (RS) participants.

**
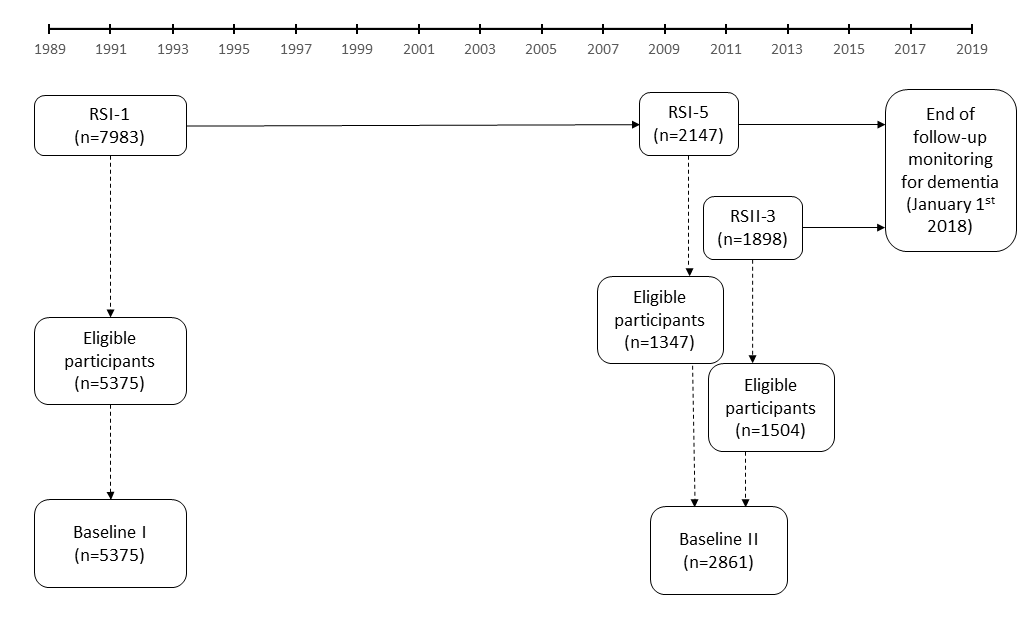
**

Table S1. Food items of the food frequency questionnaires (FFQs) that summarize the individual food components emphasized by the MIND diet

|  | **Baseline I**  **(based on 170-item FFQ)** | **Baseline II**  **(based on 389-item FFQ)** |
| --- | --- | --- |
| **Green leafy vegetables** | Spinach, Lettuce, Green cabbage  Savoy cabbage, Oxheart cabbage  Leaf cabbage, Broad-leaved endive | Spinach, Lettuce, Cabbage |
| **Other vegetables** | Cauliflower, Broccoli, Onoins, Carrot, Tomatoes, Potato, Mushrooms, Cucumber, Leek, Rhubarb, Red cabbage, Sprouts, Chicory raw, White cabbage, Sauerkraut, Vegetable soup, Garden, Peas, Pickle, Paprika, Green beans, Beets, String beans, Additional raw vegetables | Cauliflower, Broccoli, Onoins, Carrot, Tomatoes, Potato, Additional vegetables, Additional raw vegetables |
| **Nuts** | Flax seed, Peanuts, Peanut butter*0.8, Cocktail nuts*0.6, Mixed nuts | Flax seed, Peanuts, Peanut butter*0.8, Walnuts, nuts with raisins*0.5, Additional nuts |
| **Berries** | Strawberries | Strawberries |
| **Beans** | Broad beans, white/brown beans, capuchins, soup with legumes*0.4 | Beans, legumes, soup with legumes*0.4 |
| **Whole grains** | Brown rice, muesli, oatmeal, wheat bread, whole grain bread, rye bread , wheat germ, wheat bran | Brown rice, muesli, oatmeal, wheat bread, whole grain bread, rye bread , multigrain bread, high-fiber breakfast cereals, wholemeal rusk, high fiber crispbread, whole wheat dough |
| **Fish** | Mussels, Salmon, Herring, Eel, Shrimps, Herring, Mackerel, Sardines, Plaice, codfis, additional types of fish | Mussels, Salmon, Herring, Flatfish, Frout  Toastje with fish*0.4, fish salad *0.4, additional types of fish |
| **Poultry** | Chicken | Chicken |
| **Olive oil** | Olive oil vs. other cooking oils of fats | Olive oil vs. other cooking oils of fats |
| **Wine** | Red wine, white wine | Red wine, white wine |
| **Red meat** | horse meat, mutton, corn beef, sausage, ham, beef liver, pork liver, chicken liver, brined meat, smoked beef, bacon fresh, lunch meat, black pudding, saveloy, breakfast bacon, liverwurst, casserole smoked, beef, shoulder ham, minced meat roast, sausage cooked, raw beef steak, minced beef, beef roulade, roast beef, beef ribs, beef laced lap, beef tartare, pork, bratwurst, pork fricand, pork ham steaks, tenderloin, minced meat, hamburger, roast beef, beef stew, fricandeau, pork, veal, lamb | Liverm, liverproducts, gammon, raw ham, bacon, unknown type of meat products , miced meat, beef steak, beef blind finch, pork hamlap, pork chop, pork smoked sausage, additional types of meat, organ meats, unkown type of meat, meatball, liverwurst, lunch meat |
| **Butter and stick margarine** | Cooking fat, butter, frying fat, margarine | Cooking fat, butter, frying fat, margarine, lard, unkown and additional types of fat |
| **Cheese** | Cheese 48+, Cheese 40+, Cheese 20+, Cheese spread 48+ | Cheese 48+, Cheese 40+, Cheese 20 and 30+, fat luxury cheese, Less fat luxury cheese, Unkown type of cheese |
| **Fast fried food** | Croquette, fish sticks, fried fish | Fries, snack bar products, spring roll |
| **Pastries and sweets** | Cake, cookie, pancake, whipped cream, ice cream, candies, sugar, custard vanilla full, full chocolate milk, semi-skimmed chocolate milk,  sweet sandwich spread, dark chocolate, chocolate bar, biscuit, vanilla ice cream,  pudding vanilla, coke, other soft drinks, apple pie, whipped cream cake, honey, jelly, syrup, licorice | Cake, cookie, pancake, whipped cream, ice cream, candies, sugar, full custard, full chocolate milk, semi-skimmed chocolate milk, unkown types of chocolate milk, chocolate spread, sweet sandwich spread, dark chocolate, milk chocolate, white chocolate, candy bar, soft drink, pie, croissants, mousse |

Table S2. Characteristics of the study population at baseline I and II stratified for MIND diet tertiles

|  | **Baseline I** | | | **Baseline II** | | |
| --- | --- | --- | --- | --- | --- | --- |
|  | **Tertile 1**  **2.0-5.0**  **(N=1683)** | **Tertile 2**  **5.5-6.5**  **(N=2321)** | **Tertile 3**  **7.0-11.5**  **(N=1371)** | **Tertile 1**  **1.5-6.5**  **(N=1031)** | **Tertile 2**  **7.0-7.5**  **(N=681)** | **Tertile 3**  **8.0-13.5**  **(N=1149)** |
| **Sex** (women)  **Age** (years)  **Education attainment**  Primary  Lower  Intermediate  Higher  **Smoking status**  Never  Former  Current  **Body mass index** (kg/m^2^)  **Physical activity** (MET h/week)^a^  **Daily energy intake** (kcal)  **Diabetes** (yes)  **Hypercholesterolemia** (yes)  **Hypertension** (yes)  **Depressive symptoms** (yes)  **History of stroke** (yes)  ***APOE* ɛ4 carrier** (yes)  **Diet scores, mean ± standard deviation**  **MIND diet score**  **Dutch dietary guidelines score**  **Mediterranean diet score** | 884 (52.4)  68.6 ± 8.0  390 (23.3)  708 (42.1)  460 (27.3)  117 (7.0)  482 (28.6)  654 (38.9)  482 (28.6)  26.3 ± 3.6  79.5 ± 43.6  2099 ± 509  119 (7.1)  1083 (64.3)  985 (58.5)  276 (16.4)  40 (2.4)  462 (27.4)  4.5 ± 0.6  5.8 ± 1.6  34.9 ± 3.2 | 1385 (59.7)  67.8 ± 7.7  495 (21.3)  981 (42.3)  639 (27.5)  194 (8.4)  785 (33.85)  1022 (44.0)  499 (21.5)  26.3 ±3.6  82.4 ± 43.7  1961 ± 483  145 (6.2)  1561 (67.3)  1423 (61.3)  348 (15.0)  32 (1.4)  606 (26.1)  6.0 ± 0.4  6.9 ± 1.6  36.8 ± 3.0 | 900 (65.6)  66.2 ± 7.4  217 (15.8)  592 (43.2)  405 (29.5)  152 (11.1)  476 (34.7)  620 (45.2)  266 (19.4)  26.4 ± 3.7  89.1 ± 44.1  1841 ± 476  93 (6.8)  958 (69.9)  794 (57.9)  171 (12.5)  26 (1.9)  348 (25.3)  7.6 ± 0.7  7.8 ± 1.7  48.5 ± 3.0 | 544 (52.8)  76.0 ± 6.3  80 (7.8)  458 (44.4)  341 (33.1)  135 (13.1)  297 (28.8)  610 (59.2)  124 (12.0)  27.5 ± 4.3  44.4 ± 43.4  1953 ± 670  152 (14.7)  560 (54.3)  889 (86.2)  269 (26.1)  62 (6.0)  265 (25.7)  5.7 ± 0.8  5.8 ± 1.6  34.6 ± 3.8 | 383 (56.2)  75.5 ± 5.7  40 (5.9)  310 (45.5)  223 (32.7)  99 (14.5)  226 (33.2)  399 (58.6)  54 (7.9)  27.5 ± 4.0  50.6 ± 46.5  1996 ± 650  104 (15.3)  358 (52.6)  589 (86.5)  166 (24.4)  34 (5.0)  153 (22.5)  7.2 ± 0.2  6.8 ± 1.6  37.5 ± 3.4 | 716 (62.3)  74.6 ± 5.5  72 (6.3)  447 (38.9)  366 (31.9)  235 (20.5)  391 (34.0)  658 (57.3)  96 (8.4)  27.4 ± 4.2  54.6 ± 45.9  2031 ± 649  162 (14.1)  631 (54.9)  983 (85.6)  278 (24.2)  49 (4.3)  285 (22.8)  9.0 ± 1.0  7.7 ± 1.7  39.2 ± 3.5 |

Note: Data are shown for non-imputed data and are presented as mean ± standard deviation for continuous variables and number (percentages) for categorical variables. Abbreviations: *APOE*, apolipoprotein ɛ; MET, Metabolic Equivalent of Task; MIND, Mediterranean- Dietary Approaches to Stop Hypertension Intervention for Neurodegenerative Delay; N, total number of participants

Table S3. Characteristics of the study population at baseline I and II stratified for age above and below 75 years

|  | **Baseline**  **I**  **(between 1989-1993)** | | **Baseline II**  **(between 2009-2012)** | |
| --- | --- | --- | --- | --- |
|  | **Age <75 years**  **(n=4337)** | **Age ≥75 years**  **(n=1039)** | **Age <75 years**  **(n=1506)** | **Age ≥75 years**  **(n=1355)** |
| **Sex** (women) | 2501 (57.7) | 668 (64.4) | 875 (58.1) | 768 (56.7) |
| **Age** (years) | 64.8 ± 5.4 | 79.5 ± 3.7 | 70.8 ± 2.5 | 80.4 ± 4.1 |
| **Education attainment** |  |  |  |  |
| Primary | 723 (16.8) | 379 (36.7) | 64 (4.4) | 128 (9.5) |
| Lower | 1900 (44.0) | 381 (36.8) | 649 (44.4) | 566 (42.1) |
| Intermediate | 1288 (29.8) | 216 (20.9) | 453 (31.0) | 477 (35.5) |
| Higher | 405 (9.4) | 58 (5.6) | 297 (20.3) | 172 (12.8) |
| **Smoking status** |  |  |  |  |
| Never | 1309 (30.4) | 491 (47.5) | 493 (32.8) | 421 (31.2) |
| Former | 1910 (44.3) | 381 (36.8) | 840 (55.9) | 827 (61.2) |
| Current | 1091 (25.3) | 379 (36.7) | 171 (11.4) | 103 (7.6) |
| **Body mass index** (kg/m^2^) | 26.3 ± 3.6 | 26.5 ± 3.8 | 27.6 ± 4.2 | 27.4 ± 4.2 |
| **Physical activity** (MET h/week) | 86.9 ± 43.8 | 58.9 (36.7) | 57.3 ± 46.9 | 41.4 ± 42.0 |
| **Daily energy intake** (kcal) | 1989 ± 511 | 1910 (442) | 2012 ± 641 | 1975 ± 676 |
| **Diabetes** (yes) | 256 (6.1) | 101 (10.0) | 216 (14.8) | 202 (15.7) |
| **Hypercholesterolemia** (yes) | 2973 (68.6) | 629 (60.7) | 831 (56.6) | 718 (55.6) |
| **Hypertension** (yes) | 2409 (55.7) | 793 (76.8) | 1233 (83.0) | 1228 (91.4) |
| **Depressive symptoms** (yes) | 241 (16.6) | 73 (32.3) | 199 (13.6) | 231 (18.0) |
| **History of stroke** (yes) | 59 (1.4) | 39 (3.8) | 55 (3.7) | 90 (6.6) |
| ***APOE* ɛ4 carrier** (yes) | 1159 (18) | 257 (25.8) | 390 (27.6) | 213 (24.5) |
| **Diet scores, mean ± standard deviation (range)** |  |  |  |  |
| **MIND diet** | 6.0 ± 1.3 (2.0-11.5) | 5.7 ± 1.2 (2.5-10.5) | 7.5 ± 1.7 (1.5-13.5) | 7.2 ± 1.6 (2.5-12.0) |
| **Dutch dietary guidelines** | 6.8 ± 1.8 (1.0-13.0) | 6.8 ± 1.7 (1.0-11.0) | 6.8 ± 1.8 (1.0-12.0) | 6.8 ± 1.8 (2.0-13.0) |
| **Mediterranean diet** | 36.7 ± 3.4 (20.0-47.0) | 36.3 ± 3.2 (23.0-46.0) | 37.2 ± 4.1 (21.0-51.0) | 37.0 ± 4.1 (19.0-49.0) |

Note: Data are shown for non-imputed data and are presented as mean ± standard deviation for continuous variables and number (percentages) for categorical variables. Abbreviations: *APOE*, apolipoprotein ɛ; MET, Metabolic Equivalent of Task; MIND, Mediterranean- Dietary Approaches to Stop Hypertension Intervention for Neurodegenerative Delay; N, total number of participants

Table S4. Adherence scores of the individual food components

|  | **Adherence score at baseline I** | | | | **Adherence score at baseline II** | | | |
| --- | --- | --- | --- | --- | --- | --- | --- | --- |
|  | **No**  **(%)** | **Moderate**  **(%)** | **Good**  **(%)** | **Mean** | **No**  **(%)** | **Moderate**  **(%)** | **Good**  **(%)** | **Mean** |
| **Green leafy vegetables** | 73.9 | 24.1 | 2.0 | 0.1 | 71.8 | 20.8 | 7.4 | 0.2 |
| **Other vegetables** | 0.6 | 1.2 | 98.2 | 1.0 | 9.0 | 11.3 | 79.7 | 0.9 |
| **Nuts** | 52.3 | 37.4 | 10.4 | 0.3 | 27.2 | 59.3 | 13.5 | 0.4 |
| **Berries** | 85.5 | 11.6 | 3.0 | 0.1 | 45.4 | 21.7 | 33.0 | 0.4 |
| **Beans** | 51.0 | 47.3 | 1.6 | 0.3 | 15.8 | 54.6 | 29.6 | 0.6 |
| **Whole grains** | 13.8 | 67.7 | 18.6 | 0.5 | 21.2 | 68.2 | 10.6 | 0.4 |
| **Fish** | 40.0 | 19.9 | 40.1 | 0.5 | 13.0 | 18.9 | 68.1 | 0.8 |
| **Poultry** | 60.6 | 31.9 | 7.5 | 0.2 | 54.8 | 28.6 | 16.5 | 0.3 |
| **Olive oil** | 99.0 | - | 1.0 | 0.0 | 91.1 | - | 8.9 | 0.1 |
| **Wine** | 66.9 | 30.8 | 2.4 | 0.2 | 56.5 | 42.3 | 1.3 | 0.2 |
| **Red meat** | 39.3 | 41.8 | 19.0 | 0.4 | 23.8 | 36.9 | 39.4 | 0.6 |
| **Butter and stick margarine** | 20.8 | 22.2 | 57.0 | 0.7 | 10.7 | 26.3 | 63.0 | 0.8 |
| **Cheese** | 55.5 | 39.1 | 5.5 | 0.2 | 33.4 | 57.0 | 9.6 | 0.4 |
| **Fast fried food** | 0.2 | 5.9 | 93.9 | 1.0 | 8.7 | 33.8 | 57.5 | 0.7 |
| **Pastries and sweets** | 51.8 | 14.1 | 34.1 | 0.4 | 40.9 | 15.8 | 43.3 | 0.5 |

Note: If participants used olive oil as primary cooking fat (>50%) a 1 was assigned (good adherence) and a 0 otherwise (no adherence). For each other food component, a 0 was assigned if participants did not adhere to the recommendations, a 0.5 for moderate adherence, and a 1 for good adherence
